# A71 POLYP TO ADENOMA CONVERSION FACTOR AS A SURROGATE FOR ADENOMA DETECTION RATE-– FINDINGS FROM THE SOUTHWEST ONTARIO COLONOSCOPY COHORT

**DOI:** 10.1093/jcag/gwab049.070

**Published:** 2022-02-21

**Authors:** S Alobaid, C Mcdonald, L Guizzetti, B Yan, V Jairath, M Sey

**Affiliations:** 1 Gastroenterology, University of Ottawa Faculty of Medicine, Ottawa, ON, Canada; 2 Lawson Health Research Institute, London, ON, Canada; 3 Medicine, Western University, London, ON, Canada; 4 Western University, London, ON, Canada; 5 Independent researcher, London, ON, Canada

## Abstract

**Background:**

The adenoma detection rate (ADR) is one of the main quality indicators of a colonoscopy but requires combining endoscopic and histologic data. However, the polyp detection rate (PDR) requires only endoscopic assessment and has been proposed as a proxy measure for the ADR.

**Aims:**

To calculate a conversion factor for PDR to ADR, for use as a future surrogate of ADR when only PDR is available.

**Methods:**

The Southwest Ontario Colonoscopy cohort consists of all outpatient colonoscopies performed across 20 hospitals in Southwestern Ontario between April 2017 and February 2018. Data was collected prospectively through a mandatory quality assurance form that was completed after each procedure and pathology reports were manually reviewed. Endoscopies with associated histologic findings were included.

The PDR and true ADR were calculated for each physician. A weighted polyp to adenoma detection rate quotient (APDRQ) was calculated, weighting each physician’s APDRQ by the number of procedures performed. The APDRQ was determined for all outpatient procedures and specifically for screening/surveillance indications.

**Results:**

During the study period, 57 endoscopists performed 31,721 colonoscopies. The overall PDR was 41.1% and the ADR was 26.5%. The weighted ADPDRQ was 0.638 (95% CI: 0.600, 0.675). When limited to screening/surveillance colonoscopies, the weighted ADPDR was 0.616 (95% CI: 0.564, 0.669). To better understand the influence of endoscopists with low ADR: PDR, we excluded those with ratio below (<2 standard errors) the average, which resulted in greater ADR: PDR for all colonoscopies 0.695 (95% CI: 0.679, 0.711) and for screening/surveillance colonoscopies and 0.692 (95% CI: 0.677, 0.707).

**Conclusions:**

In this large, population-based, cohort study, we calculated the ADR; PDR ratio. We propose this may be used in future studies to infer ADR when only PDR is available.

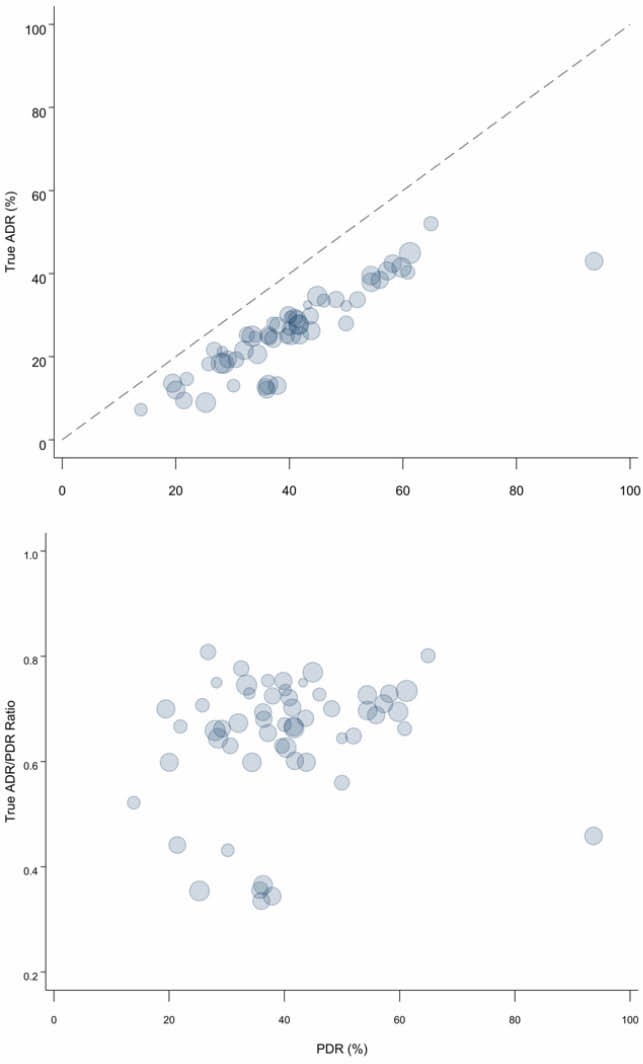

Scatter plot of correlation between ADR and PDR, by physician. The dashed line indicates the line for which ADR=PDR, the maximum value the ADR can take for a given PDR. The marker size is proportional to the number of colonoscopies performed.

**Funding Agencies:**

None

